# Early-onset autoimmune disease due to a heterozygous loss-of-function mutation in *TNFAIP3* (A20)

**DOI:** 10.1136/annrheumdis-2016-210944

**Published:** 2017-06-28

**Authors:** Christopher J A Duncan, Emma Dinnigan, Rachel Theobald, Angela Grainger, Andrew J Skelton, Rafiqul Hussain, Joseph D P Willet, David J Swan, Jonathan Coxhead, Matthew F Thomas, Julian Thomas, Veena Zamvar, Mary A Slatter, Andrew J Cant, Karin R Engelhardt, Sophie Hambleton

**Affiliations:** 1 Primary Immunodeficiency Group, Institute for Cellular Medicine, Newcastle University, UK; 2 Bioinformatics Support Unit, Institute for Cellular Medicine, Newcastle University, UK; 3 Genomic Core Facility, Institute for Genetic Medicine, Newcastle University, UK; 4 Department of Paediatric Respiratory Medicine, Great North Children’s Hospital, Royal Victoria Infirmary, UK; 5 Department of Paediatric Gastroenterology, Great North Children’s Hospital, Royal Victoria Infirmary, UK; 6 Department of Paediatric Gastroenterology, Leeds General Infirmary, UK; 7 Department of Paediatric Immunology and Stem Cell Transplant Unit, Great North Children’s Hospital, Newcastle upon Tyne Hospitals NHS Foundation Trust, UK

**Keywords:** Complex autoimmunity, Primary immunodeficiency, NF-κB regulation, Monogenic autoimmunity, TNF signalling, Immune homeostasis, A20 haploinsufficiency

Rare Mendelian disorders increasingly contribute to our understanding of the genetic architecture of autoimmune disease and the key molecular pathways governing its pathogenesis. Early-onset autoimmune disease can arise through activating mutations in inflammatory signalling pathways or loss-of-function mutations in immunoregulatory proteins.

We investigated the molecular basis of complex autoimmunity—characterised by the onset of insulin-dependent diabetes, cytopaenias, hepatitis, enteropathy and interstitial lung disease at age 10—in a 14-year-old boy of healthy non-consanguineous British parents. Immunological analysis revealed lymphopaenia with no naive T cells and a high proportion of activated T cells (table 1). Pathogenic variants in *STAT3* and *FOXP3* were excluded. The clinical course was refractory to intensive immunosuppression with prednisolone, sirolimus, tacrolimus, infliximab or rituximab, necessitating haematopoietic stem cell transplantation. Twenty-one months post-transplant, he is thriving off all immunosuppressive medication with complete remission of autoimmune disease (except diabetes).

**Table 1:** Immunological and clinical parameters

Parameters	Pretransplant	Post-transplant	Reference range
Laboratory			
Haemoglobin (g/dL)	9.8	12.4	13.5–17.5
Leucocytes (10^9^/L)	1.88	3.47	150–450
Lymphocytes (10^9^/L)	0.17	1.31	1.2–5.2
Neutrophils (10^9^/L)	1.52*	1.79	1.8–8.0
Monocytes (10^9^/L)	0.19	0.37	0.2–0.8
Platelets (10^9^/L)	29	183	150–400
CD3+ (cells/µL)	800	1914	800–3500
CD8+ (cells/µL)	554	936	200–1200
CD4+ (cells/µL)	238	920	400–1200
CD56+ (cells/µL)	35	99	70–1200
CD19+ (cells/µL)	138	99	200–600
Activated T cells (HLA-DR+ %)	55	25	N/A
CD4+ naive (%)	Not detected	244	N/A
CD27– IgD+ (naive) (%)	87	93	75.2–86.7
CD27+ IgD+ (memory) (%)	9	4	4.6–10.2
CD27+ IgD– (class-switched) (%)	2	3	3.3–9.6
IgM (g/L)	0.55	0.25	0.50–1.90
IgG (g/L)	6.4	8.2	5.4–16.1
IgA (g/L)	0.92	0.33	0.80–2.80
Tetanus (IU/mL)	0.93	ND	0.1–10
*Haemophilus influenzae* b (mg/mL)	1.8	ND	1.0–20.0
Pneumococcal (mg/mL)	10	ND	20–200
Anti-GAD antibody (IU/mL)	>2000	>2000	0–9.9
Islet cell antibody	Detected	Detected	N/A
pANCA	Detected	Detected	N/A
Clinical			
FEV_1_ (% predicted)	38	84	95–100

*Peripheral neutrophils were supported pretransplant by recombinant granulocyte colony stimulating factor. Post-transplant parameters were obtained at 18 months (FBC and T-cell indices, lung function) or 21 months post-HSCT (B cell and antibody indices). Post-HSCT antibody indices were measured during concomitant subcutaneous immunoglobulin supplementation. No other autoantibodies were detected pre-HSCT or post-HSCT.

FBC, full blood count; FEV_1_, forced expiratory volume in 1 s; GAD, glutamic acid decarboxylase; HLA-DR, human leucocyte antigen–antigen D related; HSCT, haematopoietic stem cell transplantation; ND, not done; pANCA, perinuclear anti neutrophil cytoplasmic antibody.

**Figure 1:**
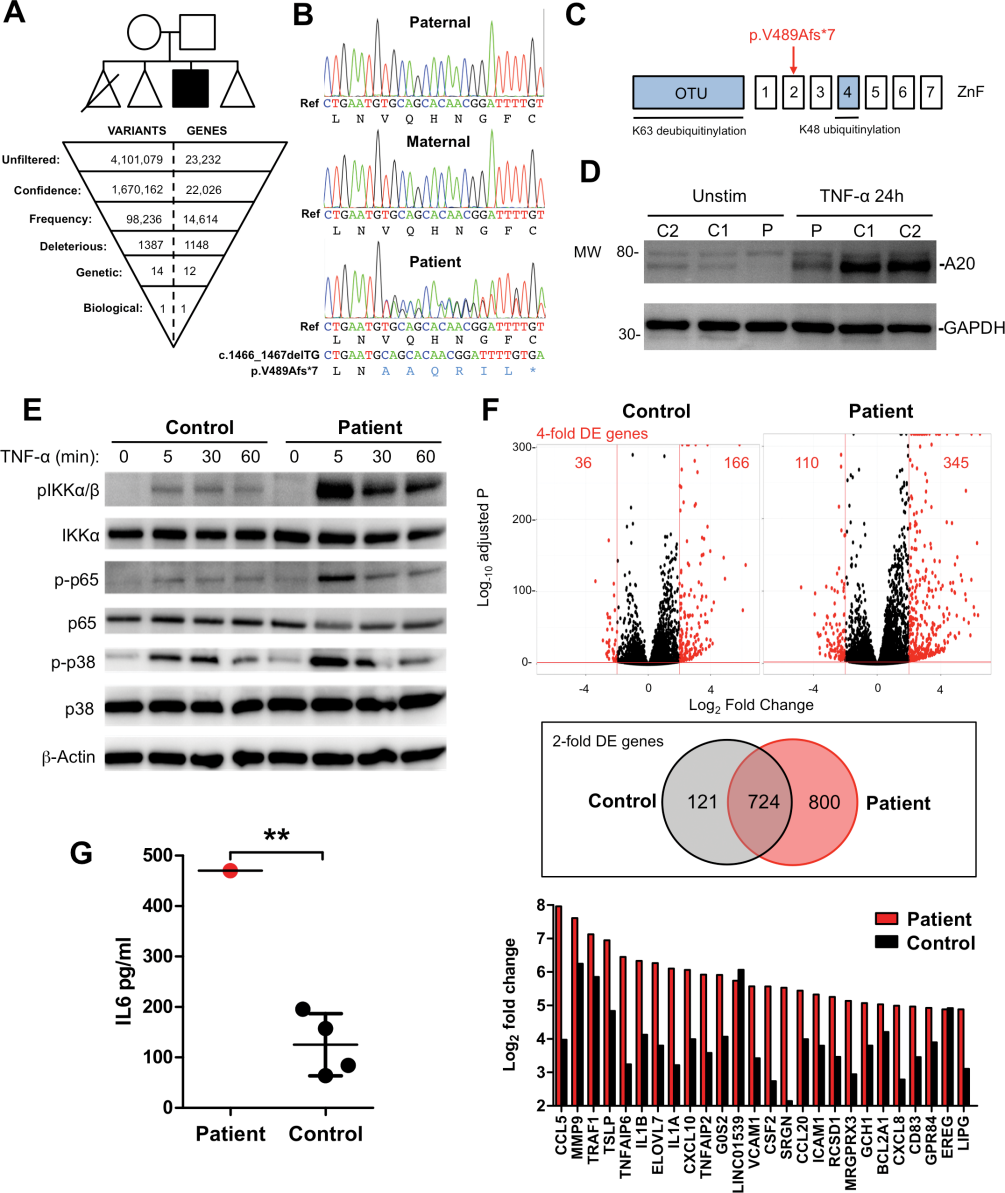
*TNFAIP3* variant identification and functional validation. (**A**) The family pedigree is shown (triangles are used to preserve the anonymity of healthy unaffected siblings). The first-born infant died as a result of prematurity. Whole exome sequencing data were filtered (Ingenuity Variant Analysis) by confidence (call quality ≥20; read depth ≥10; allele fraction ≥45%); frequency (ExAc allele frequency ≤0.01%); deleteriousness (nonsense/deleterious missense (SIFT/PolyPhen), splice-site disruption); genetic segregation (ie, present in patient and absent from 47 unrelated disease controls) and biological function (linked to phenotype), identifying a single heterozygous frameshift variant in *TNFAIP3* (c.1466_1467TGdel). (**B**) Variant confirmation by Sanger sequencing. (**C**) The c.1466_1467TGdel variant resulted in a frameshift and premature stop codon (V489Afs*7) in the second ZnF domain and is distinct from previously described mutations in the OTU and ZnF4 domains (blue). (**D**) V489Afs*7 reduced basal and TNF-induced A20 protein in patient (P) versus control (C1, C2) fibroblasts (immunoblot representative of n=4 independent experiments with n=4 controls). (**E**) Signalling responses downstream of TNF-α stimulation in patient fibroblasts were exaggerated and prolonged compared with control (immunoblot representative of n=4 independent experiments with n=4 controls). (**F**) RNA-seq analysis of transcriptional response to 6-hour TNF-α stimulation in patient and control fibroblasts (stimulations performed in triplicate in a single experiment). Top panel: displayed in red are significant (FDR-corrected p≤0.01) DE transcripts regulated ≥4 fold (≥2log_2_-fold); middle panel: Venn diagram displaying all overlapping DE transcripts ≥2 fold (≥log_2_-fold); Bottom panel: top 20 significant DE transcripts in patient (red bars) versus control (black bars), demonstrating many major NF-κB target genes. (**G**) Levels of IL-6 quantified by ELISA in supernatants from patient and control fibroblasts stimulated with TNF-α for 24 hours (mean±SD of average values from two independent experiments in patient and n=4 controls compared by one-sample t-test; **p=0.0015). DE, differentially expressed; FDR, false discovery rate; IL-6, interleukin 6; NF-κB, nuclear factor-κB; OTU, ovarian tumour; PolyPhen, polymorphism phenotyping; SIFT, Sorting Intolerant from Tolerant; TNF-α, tumour necrosis factor-alpha; TNFAIP3, tumour necrosis factor-alpha-induced protein 3; ZnF, zinc finger.

Ethical approval was granted (ref: 10/H0906/22) and written informed consent provided prior to study commencement. By whole exome sequencing of peripheral blood genomic DNA (Illumina MiSeq) and downstream bioinformatic filtering (Ingenuity Variant Analysis), we identified a single biologically plausible variant—a novel de novo heterozygous 2 bp deletion in tumour necrosis factor-alpha-induced protein 3 (*TNFAIP3*, figure 1A). *TNFAIP3* encodes the ubiquitin-editing enzyme A20, a negative regulator of the nuclear factor-κB (NF-κB) pathway.^1^ A20 removes K63-linked ubiquitin chains from key adaptor proteins, replacing them with K48-linked polyubiquitin chains, to trigger proteasomal degradation and termination of the NF-κB activation cascade.^2^ Polymorphisms in *TNFAIP3* have been linked to the development of several autoimmune diseases in genome-wide association studies.^3–7^ A conditional knockout of A20 in immune cells leads to the development of autoimmunity in the mouse.^8^ However, autoimmune phenomena were not prominent in a recently described cohort of patients with germline A20 haploinsufficiency, who instead presented with an autoinflammatory phenotype resembling Behçet’s disease.^9^

The c.1466_1467delTG variant—which we confirmed by capillary sequencing^10^ (figure 1B)—introduces a frameshift substitution of alanine for valine at position 489, generating a downstream premature stop codon (p.V489Afs*7) in the zinc finger (ZnF)2 domain of A20. This variant is absent from public databases (ExAc/dbSNP) and distinct from disease-associated mutations affecting the ovarian tumour or ZnF4 domains of A20^9^ (figure 1C). Immunoblotting^10^ of patient and control dermal fibroblast lysates with an N-terminal antibody confirmed the reduced basal and TNF-α-induced expression of A20 (figure 1D).

To address the consequence of this reduced A20 expression, we performed functional experiments in patient and control dermal fibroblasts. Initially, we stimulated these cells with TNF-α (10 ng/mL) and analysed downstream signalling events by immunoblot (figure 1E). We observed exaggerated and prolonged phosphorylation of components of the NF-κB pathway, which would be expected to enhance NF-κB-dependent transcriptional effects. In keeping with this prediction, RNA sequencing (Illumina NextSeq-500) revealed a significant global increase in both the range and magnitude of TNF-α-stimulated differential gene expression (fold-change ≥2; false discovery rate-adjusted p≤0.01, figure 1F). We also confirmed enhanced expression of the key NF-κB target gene interleukin 6 (IL-6) at the protein level by ELISA (p=0.0015, figure 1G). In these respects, the molecular consequences of the p.V489Afs*7 variant were indistinguishable from reported pathogenic A20 mutations,^9^ although owing to the lack of leucocyte material, we were not able to extend our analysis to inflammasome activation.

Here we provide novel validation of considerable existing evidence that implicates *TNFAIP3* in autoimmune pathogenesis. This case expands the clinical spectrum of A20 haploinsufficiency.^9^ As A20 regulates multiple innate and adaptive signalling pathways,^1^ it is logical that patients with inactivating mutations in A20 might manifest pathological features of autoimmunity and/or autoinflammation. Finally, we report that correction of the molecular defect within the haematopoietic cell compartment could represent a viable treatment option for severe clinical manifestations.
